# Linking Phylogeny and Morphology to Resource Assimilation Within Aquatic Assemblages

**DOI:** 10.1002/ece3.70641

**Published:** 2024-11-24

**Authors:** Matthew B. Lodato, Brian C. van Ee, Carla L. Atkinson

**Affiliations:** ^1^ Department of Biological Sciences University of Alabama Tuscaloosa Alabama USA

**Keywords:** cilia per cirrus, cirri density, freshwater mussels, phylogenetic distance, trophic niche

## Abstract

Niche partitioning promotes species coexistence. Yet, it remains unclear how phylogeny and morphology influence the trophic niches of closely related aquatic species with shared feeding modes. Freshwater mussels (Family: Unionidae) are a group of filter‐feeding bivalves that are ideal for investigating mechanisms of niche partitioning. Particle size selection and patterns of ingestion are controlled by gill latero‐frontal cirri density (CD) and the number of cilia per cirrus (CC). We investigated trophic assimilation and niche area using stable isotope signatures (𝛿^13^C and 𝛿^15^N) and gill morphology with scanning‐electron microscopy for a diverse mussel assemblage from the Sipsey River, Alabama, USA. We predicted that (1) trophic niches and gill morphology would differ within and among species across sites; (2) co‐occurring species would partition food resources; (3) greater phylogenetic distances among species would result in increased trophic dissimilarity; (4) more CC and higher CD would result in a narrower trophic niche area, or more constrained range of food items assimilated. We found that (1) species identity and site influenced gill morphology and stable isotope signatures but that the trophic niche area of a species was only affected by species identity; (2) the average proportion of niche area overlap between co‐occurring species was low across sites (0.04 to 0.18); (3) trophic dissimilarity among species increased with phylogenetic distance; (4) CD but not the number of CC negatively related to trophic niche area. Our results indicate that gill morphology and evolutionary history are likely key factors governing the trophic niches of mussels. In addition, intraspecific variation in gill morphology across sites may either reflect a phenotypic response to differences in local resource availability or suggest that other mechanisms shape particle selection. Examining the interplay among the trophic niche, phylogeny, and morphology among functionally similar species further informs our understanding of the mechanisms facilitating their coexistence.

## Graphical abstract

We examined how phylogeny and morphology affected resource assimilation patterns within aquatic assemblages, specifically freshwater mussels (Family: Uniondae). We found that (1) species identity and site influenced gill morphology and stable isotope signatures but that the trophic niche area of a species was only affected by species identity; (2) the average proportion of niche area overlap between co‐occurring species was low across sites (0.04 to 0.18); (3) trophic dissimilarity among species increased with phylogenetic distance; (4) latero‐frontal cirri density but not the number of cilia per cirrus negatively related to trophic niche area. Our results indicate that evolutionary history and gill morphology are likely key factors governing the trophic niche of mussels.

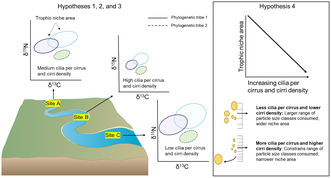

## 1 Introduction

Understanding mechanisms that promote species coexistence has been a longstanding topic in ecology (Hutchinson 1959). The niche represents the environmental conditions and resources required by a species (Hutchinson 1959), and niche complementarity within communities is an important mechanism that underlies the evolution of trait diversification (MacArthur 1958; Pigot et al. 2018; Schoener 1974). Niche partitioning is governed by species traits that enable community members to adapt to varying environmental conditions and can include differences in physiological tolerances (Hilton, Wellenreuther, and Clements 2008), spatial and temporal patterns in habitat use (Nakano, Fausch, and Kitano 1999), and body morphology (Winemiller 1991). Understanding the mechanisms promoting niche partitioning is important because it governs biodiversity (Levine and HilleRisLambers 2009) and enhances ecosystem stability and function (Cardinale 2011; Chesson 2000).

The trophic niche, or range of food items consumed by a species (Bearhop et al. 2004), is commonly characterized using stable isotope analysis as it provides time‐integrated measurements of the food items assimilated by community members (Layman et al. 2007). Specifically, δ^13^C is indicative of the relative proportion of basal resource types while δ^15^N represents trophic position because it increases relatively predictably with trophic level (Post 2002). Quantification of the total trophic niche area in isotopic space can be used to characterize the degree of resource partitioning and overlap among species (Jackson et al. 2011). Trophic niche partitioning is dependent on the traits associated with co‐occurring species as these can promote food‐resource partitioning and consequently influence niche occupancy (e.g., Premate et al. 2021). Because previous research on trophic niches in aquatic systems has primarily focused on characterizing taxonomic groups with disparate feeding modes (Flood, Loftus, and Trexler 2023; Winemiller 1991), our understanding of trophic niche occupancy patterns among closely related species within the same functional feeding group is still limited (but see Sánchez González et al. 2023).

A mechanistic understanding of trophic niche partitioning can be inferred from form and function relationships, such as how morphological traits relate to the niche (Figueira et al. 2023; Premate et al. 2021; Winemiller 1991). Freshwater mussels (Family: Unionidae) are an ideal model system to assess how morphology relates to trophic assimilation. These sedentary filter‐feeders often thrive in diverse and dense aggregations that are patchily distributed throughout river networks (Haag 2012). Specifically, the Ambleminae subfamily is a species‐rich group of North American mussels (~300 species; Haag 2012) comprised of five phylogenetic tribes (Pfeiffer, Breinholt, and Page 2019). These tribes are stoichiometrically distinct and exhibit varying patterns of species diversification (Atkinson, van Ee, and Pfeiffer 2020; Pfeiffer, Breinholt, and Page 2019). This diversity, coupled with stoichiometric variation, dispersal limitations, and similarities in feeding behaviors, creates a high potential for food‐resource overlap and consequential partitioning among individuals within an aggregation (e.g., Atkinson et al. 2010; Sánchez González et al. 2023; Tran and Ackerman 2019). Furthermore, rivers exhibit tremendous spatial and temporal variation in resource quantity and quality (Atkinson et al. 2009; Vannote et al. 1980), which may also influence morphological traits and trophic niches of populations.

**FIGURE 1:**
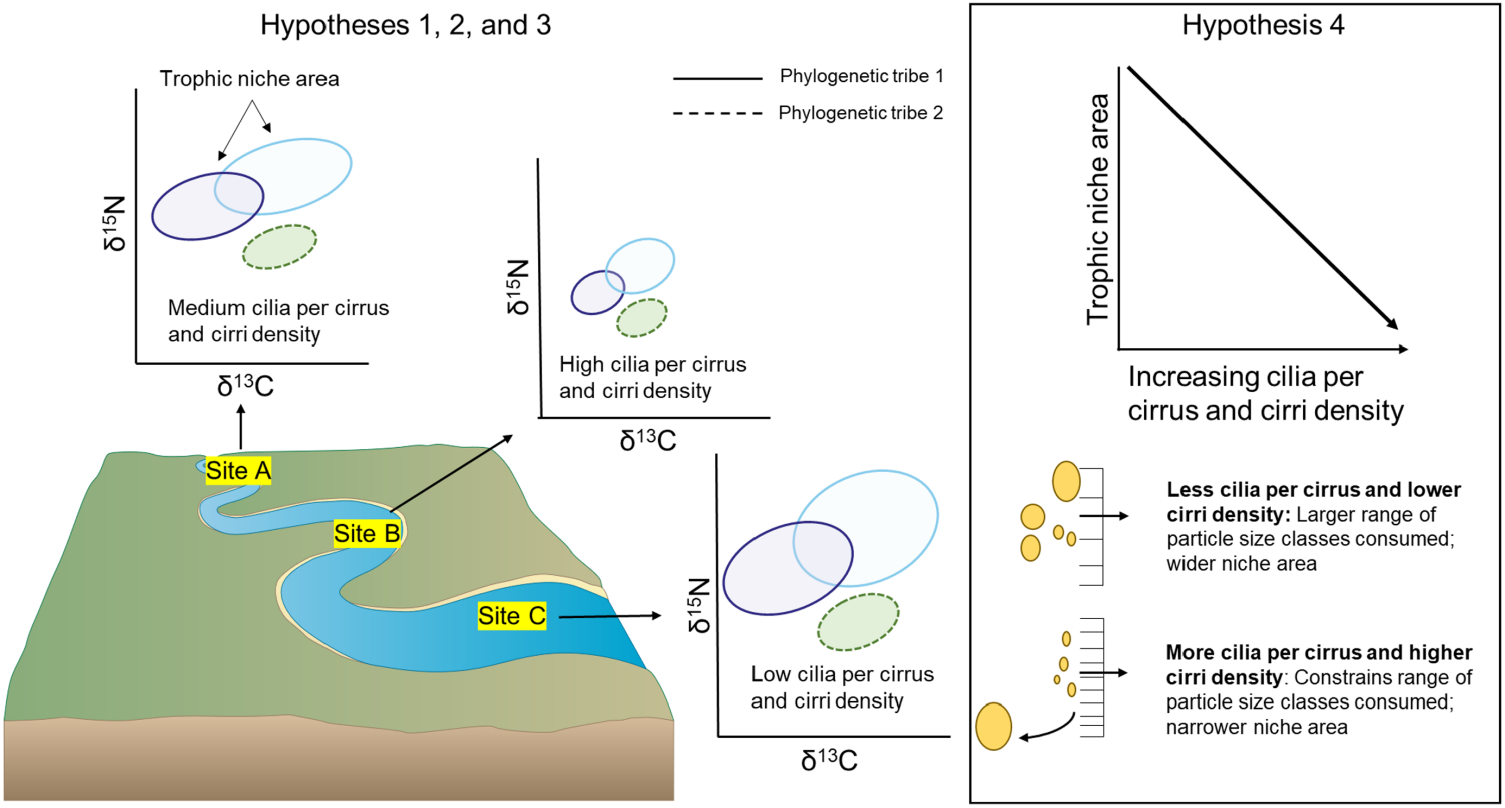
Conceptual diagram depicting the four study hypotheses: (H1) Trophic assimilation and gill morphology will vary within and among species across sites; (H2) There will be low trophic niche overlap among co‐occurring mussel species, indicating food‐resource partitioning; (H3) There will be a positive relationship between the phylogenetic distance and trophic dissimilarity of species due to increased divergence in assimilation and soft‐tissue stoichiometry; (H4) The number of cilia per cirrus and cirri density will negatively relate to the trophic niche area because less cilia per cirrus and lower densities should allow more particle size classes to be ingested. Colored ellipses represent species trophic niches. Ellipse outlines represent phylogenetic tribes. Yellow circles represent food particles interacting with latero‐frontal cirri. River graphic retrieved and modified from the Maryland Image Library (2023; ian.umces.edu/media‐library).

Mussels feed by waving gill cilia to siphon and consume particles that are suspended in the water column (Gardiner, Silverman, and Ditez 1991; Jørgensen 1990). The range of particles consumed and/or assimilated by a species may vary due to differences in life history (growth rates; Atkinson, van Ee, and Pfeiffer 2020), or the size and composition of available food particles (e.g., algae, bacteria, and detritus; Angradi 1996; Atkinson et al. 2009). Consequently, partitioning among co‐occurring mussel species may arise from selective filtration, ingestion, and assimilation of specific food particles in varying proportions. One hypothesized mechanism underlying particle selection is variation in gill morphology (Evan Ward and Shumway 2004; Riisgård and Larsen 2010). Specifically, variations in the densities of latero‐frontal cirri associated with the gills, or fused pairs of cirral plates composed of cilia, and the number of cilia per latero‐frontal cirrus (Silverman et al. 1997). More cilia per cirrus (CC) and higher cirri density (CD) are suggested to correspond to higher clearance of smaller particles (Silverman et al. 1995, 1997), and preferential rejection of larger particles over smaller ones have been observed in species with complex cirri arrangements (Defossez and Hawkins 1997). Therefore, increased numbers of CC and higher CD should constrain more particle size classes and result in a narrower trophic niche area (Figure 1). Although differences in the number of CC and CD have been documented among co‐occurring mussel species (Galbraith et al. 2009), we are not aware of any studies that have examined how variations in these traits relate to the trophic niches of co‐occurring filter‐feeders, or that have evaluated how these morphological traits and the trophic niche vary among spatially separated populations within a single river system. In addition, despite recent evidence of trophic niche partitioning among co‐occurring filter‐feeders (Conti‐Jerpe, Pawlik, and Finelli 2022; Sánchez González et al. 2023), it is still unclear whether trophic dissimilarities among species within this functional feeding group vary in accordance with their phylogenetic relatedness.

We evaluated the trophic niche (position, width, and overlap in isotopic space), phylogeny, and the number of CC and CD associated with the gills to assess their influence on trophic assimilation patterns across multiple mussel species within a single river system. We asked: (1) does the trophic niche and gill morphology of mussels vary within and among species across sites; (2) are co‐occurring species partitioning food resources; (3) does phylogenetic divergence relate to trophic dissimilarity among species; (4) is there a relationship between the trophic niche area of a species and the number of CC and CD associated with its gills? We hypothesized (H1) patterns of trophic assimilation and gill morphology would vary within and among species across sites; (H2) there would be low trophic niche overlap among co‐occurring species, indicating food‐resource partitioning; (H3) there would be a positive relationship between phylogenetic distance and trophic dissimilarity because more closely related species should have more similar stoichiometric demands (i.e., soft‐tissue elemental ratios) and therefore, dietary requirements; (H4) Both CC and CD would be negatively related to the trophic niche area because more CC and higher densities should constrain the range of particle size classes that are ingested (Figure 1).

## 2 Methods

### 2.1 Study Site and Sample Collection

**FIGURE 2:**
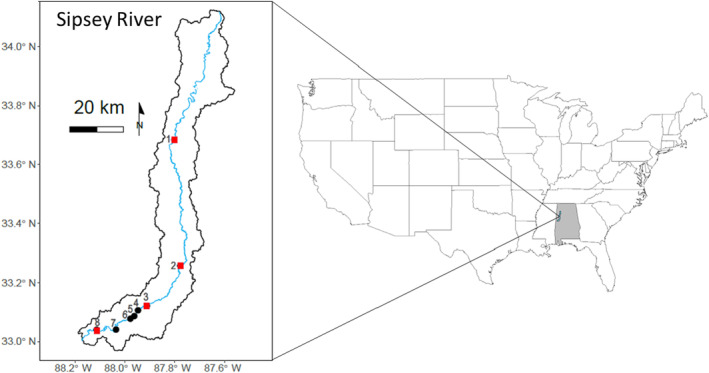
Right panel: Map of the United States with Sipsey River watershed inset in the state of Alabama (shaded gray). Left panel: Map of Sipsey River and floodplain boundary. Sites ordered upstream (Site 1) to downstream (Site 8). Red squares indicate sites with both stable isotope and gill morphology data. Black circles are sites with stable isotope data only. Site two is adjacent to Sipsey wetland complex.

We collected mussels from eight sites along the Sipsey River, AL, USA, between August and October 2016 (Appendix S1; Figure 2). The Sipsey River (watershed area 2044 km^2^) is a 5th‐order alluvial river that is relatively unmodified, harbors the majority of its historical species‐richness, and has an intact floodplain containing an extensive wetland (Atkinson et al. 2019; Haag and Warren 2010; McGregor and O'Neil 1992). Sites were positioned upstream (Site 1), adjacent to (Site 2), and downstream (Sites 3 to 8) of this wetland. We selected these sites because they contain dense and species‐rich mussel aggregations (Haag and Warren 2010; Atkinson and Forshay 2022), and resource composition varies spatially and is affected by floodplain inundation (Atkinson et al. 2019). Therefore, this is a good system to evaluate how potential variation in food availability might influence the trophic niche and gill morphology of mussels.

### 2.2 Stable Isotope Analysis

We measured the body length of 226 individuals across 11 mussel species (Table S1). Because unionid mussels are a highly imperiled animal group (Böhm et al. 2021), we limited our sample size to a maximum of five individuals per species per site. However, previous research that estimated inter‐population variability of mussel stable isotopic signatures across different sites, using five to six samples from a population, demonstrated that based on the coefficient of variation (5%), even relatively small sample sizes can produce means that are representative of larger populations (Gustafson et al. 2007). Prior to stable isotope analysis we dissected the stomachs and gastrointestinal tissues for a subset of individuals and described microbiome communities (see Weingarten, Atkinson, and Jackson 2019). Following dissection, we freeze‐dried and then homogenized the remaining soft tissue. Then, the δ^13^C, δ^15^N, %C, and %N values of remaining soft tissue were determined by elemental combustion in a Costech EA 4010 Elemental Analyzer (Costech Analytical Technologies, Valencia, CA, USA) coupled with a Thermo Delta V Plus mass spectrometer (ThermoFisher Scientific). Isotopic values were expressed in standard delta notation in per mille (‰) and were calculated using the equation: δ*X* ([*R*_sample_/*R*_standard_] − 1) × 10^3^ where *X* is the element observed and *R* is the ratio of heavy to light isotopes. The standard for δ^13^C and δ^15^N were Vienna Peedee Belemnite and atmospheric N_2_, respectively (Fry 2006). To account for differences in lipid content between individual mussels we mathematically normalized δ^13^C data using the molar C:N of the sample and the following formula (Post et al. 2007):

$$\delta^{13}C_{\text{corrected}}=\delta^{13}C_{\text{original}}-3.32+0.99\times C:N$$

### 2.3 Gill Morphology

We measured the number of gill cilia per cirrus and cirri densities for 70 individuals representing five species: *Fusconaia cerina*, *Lampsilis ornata*, *Pleurobema decisum*, *Pustulosa kieneriana*, and *Quadrula verrucosa*, across four sites: Site 1, 2, 3, and 8 (Table 1). We dissected and fixed lateral gills following modified methods from Galbraith et al. (2009). We then cut nine, 1‐cm^2^ subsamples for each individual and then captured three micrographs per gill subsample with a Hitachi Su 3500 Variable Pressure Scanning Electron Microscope (Hitachi, Chiyado City, Tokyo, Japan). Using these micrographs and ImageJ digital analysis software (Schneider, Rasband, and Eliceiri 2012), we enumerated the cilia per latero‐frontal cirrus (i.e., cilia per cirrus) and the number of latero‐frontal cirri per cm of gill filament (i.e., cirri density; Figure S1).

**TABLE 1:** Mean body length, mean number of cilia per cirrus, and mean cirri density ± standard error (SE). *N* indicates the number of mussel individuals for a species sampled within a site. NA indicates that a measurement was lost during sample collection.

Site	Species	Phylogenetic tribe	Total body length (mm) ± SE	Number of cilia per cirrus ± SE	Cirri density per cm ± SE	*N*
Site 1	*Fusconaia cerina*	Pleurobemini	52.57 (4.38)	35.7 (1.8)	5804.8 (118.8)	3
	*Lampsilis ornata*	Lampsilini	86.23 (3.38)	18.8 (1.3)	5217.2 (106.0)	4
	*Pustulosa kieneriana*	Quadrulini	50.25 (12.05)	23.4 (1.5)	4803.9 (68.1)	2
	*Quadrula verrucosa*	Quadrulini	69.58 (9.48)	16.5 (0.8)	6287.8 (148.1)	4
Site 2	*Fusconaia cerina*	Pleurobemini	54.70 (1.99)	10.2 (0.5)	4818.4 (60.6)	5
	*Lampsilis ornata*	Lampsilini	104.83 (4.74)	14.2 (0.8)	4210.3 (56.9)	4
	*Pustulosa kieneriana*	Quadrulini	52.85 (2.38)	7.9 (0.5)	4661.2 (51.1)	4
	*Quadrula verrucosa*	Quadrulini	92.05 (19.73)	13.4 (0.8)	4523.7 (60.3)	4
Site 3	*Fusconaia cerina*	Pleurobemini	44.90 (4.55)	11.4 (0.7)	5234.0 (67.4)	4
	*Lampsilis ornata*	Lampsilini	68.33 (17.08)	6.3 (0.3)	4425.9 (44.4)	3
	*Pleurobema decisum*	Pleurobemini	51.20 (4.87)	7.6 (0.4)	5612.3 (62.0)	4
	*Pustulosa kieneriana*	Quadrulini	46.37 (2.24)	14.9 (0.9)	4974.9 (66.9)	3
	*Quadrula verrucosa*	Quadrulini	NA	10.7 (0.5)	4590.0 (40.9)	5
Site 8	*Fusconaia cerina*	Pleurobemini	36.74 (2.60)	13.8 (0.7)	5131.6 (54.6)	5
	*Lampsilis ornata*	Lampsilini	79.58 (8.26)	13.7 (0.4)	4638.5 (85.1)	5
	*Pleurobema decisum*	Pleurobemini	48.05 (3.78)	12.9 (0.8)	6078.8 (38.8)	4
	*Pustulosa kieneriana*	Quadrulini	37.65 (2.73)	24.9 (1.6)	5394.4 (77.0)	4
	*Quadrula verrucosa*	Quadrulini	62.77 (4.42)	15.4 (1.0)	5247.1 (76.5)	3

### 2.4 Data Analyses

All analyses were performed using R 4.1.2 (R Core Team 2023; Wickham 2011). We used normalized δ^13^C and δ^15^N values to estimate Bayesian standard ellipse areas (SEA_B_) as a proxy for a species' trophic niche that is corrected for small sample sizes (H1; package *SIBER*; Jackson et al. 2011). In addition, estimating a species' standard ellipse area in a Bayesian framework allows for robust comparison between sample populations with unequal sample sizes (Jackson et al. 2011). Next, we estimated the proportion of SEA_B_ overlap between co‐occurring species using the maximum likelihood estimated means and covariance matrices of the ellipse area estimates associated with each species pair to evaluate resource overlap or partitioning (H2; package *SIBER*; Jackson et al. 2011). We performed Kruskal‐Wallis tests to examine the influence of site and species identity on δ^13^C, δ^15^N, and SEA_B_, respectively (H1). We used non‐parametric analysis due to considerable differences in the number of species across sites, which resulted in an unbalanced study design.

Because we were unable to collect food resources from all of our sites, we standardized soft‐tissue isotope values for species occurring at three or more sites. This process aimed to mitigate the potential influence of site‐specific variations in resources on mussel isotope signatures. Standardization involved rescaling the isotope values for each species within each site into their respective *z*‐scores. We visualized each species' trophic niche using convex hulls (Layman et al. 2007), as standard ellipses converged when generated using standardized data, leading to overlap and stacking. We tested whether species identity influenced trophic dissimilarity using a permutational analysis of variance (PERMANOVA) based on a Euclidean distance matrix generated from *z*‐scores (package *vegan*; Oksanen et al. 2020). Significant PERMANOVAs were followed by post hoc pairwise tests (Package *pairwiseAdonis*; Martínez Arbizu 2017). PERMANOVA simultaneously tests whether group centroid or dispersion differs among species. When our PERMANOVA analysis indicated that standardized isotopic signatures differed among species, we conducted a post hoc homogeneity of dispersion analysis (package *vegan*; Oksanen et al. 2020) and analyzed these results using ANOVA. Where dispersion was not different between groups, we concluded that they differed in the location of their centroids. Where dispersion was different, we concluded only that the overall isotopic signature was different and did not attempt to distinguish between differences of centroid location or group dispersion. To examine the relationship between trophic dissimilarity and phylogenetic distance among species (H3), we used the pairwise phylogenetic distance values from Atkinson, van Ee, and Pfeiffer (2020) and regressed these against the pairwise Euclidean distances calculated between species in the present study using a linear model.

We ran two‐way ANOVAs to determine the influence of site and species identity on gill morphology (H1; package *car*; Fox and Weisberg 2019). Because we only collected *Pleurobema decisum* from half of our sites due to permitting restrictions, we subdivided our data in two ways prior to analysis to maximize interspecific and intraspecific comparisons across sites. First, we excluded *P. decisum* from analysis but included the four other remaining species across Sites 1, 2, 3 and 8. Second, we included all five species but included only Site 3 and 8 in the analysis. In addition, for each data subset we performed Tukey's HSD post hoc tests to examine pairwise differences in CC and CD, respectively, between co‐occurring mussels (package *emmeans*; Lenth 2022). We used linear regression to determine if CC and CD were associated with normalized δ^13^C and δ^15^N, respectively. To examine how these traits related to the trophic niche area (SEA_B_), we used median‐based robust regression as this accounts for the influence of outliers in smaller datasets (H4; package *mblm*; Komsta 2019). Gill morphology data met the assumptions of normality and equal variance. All frequentist analyses used an alpha of 0.05.

## 3 Results

### 3.1 Stable Isotopes

Both site (*H*_7_ = 76.75; *p* < 0.0001) and species identity (*H*_10_ = 82.93; *p* < 0.0001) were related to variation in mussel δ^13^C. Notably, δ^13^C values were less depleted at Site 1 in comparison to Sites 2 through 8 (Table S1). Across sites and species, mean δ^13^C ranged from −33.44‰ ± 0.26‰ to −27.49‰ ± 0.10‰, with *Lampsilis ornata* from Site 3 having the lowest average δ^13^C signature, and *Pustulosa kieneriana* from Site 1 having the highest δ^13^C on average (±SE).

Both site (*H*_7_ = 140.19; *p* < 0.0001) and species identity (*H*_10_ = 37.85; *p* < 0.0001) influenced mussel δ^15^N, with the highest average values being associated with species from Site 2 (Table S1). Across sites and species, mean δ^15^N ranged from 5.96‰ ± 0.12‰ to 9.21‰ ± 0.21‰ (±SE). *Obovaria unicolor* from Site 7 had the lowest average δ^15^N values, while this same species from Site 2 had the highest δ^15^N on average.

**FIGURE 3:**
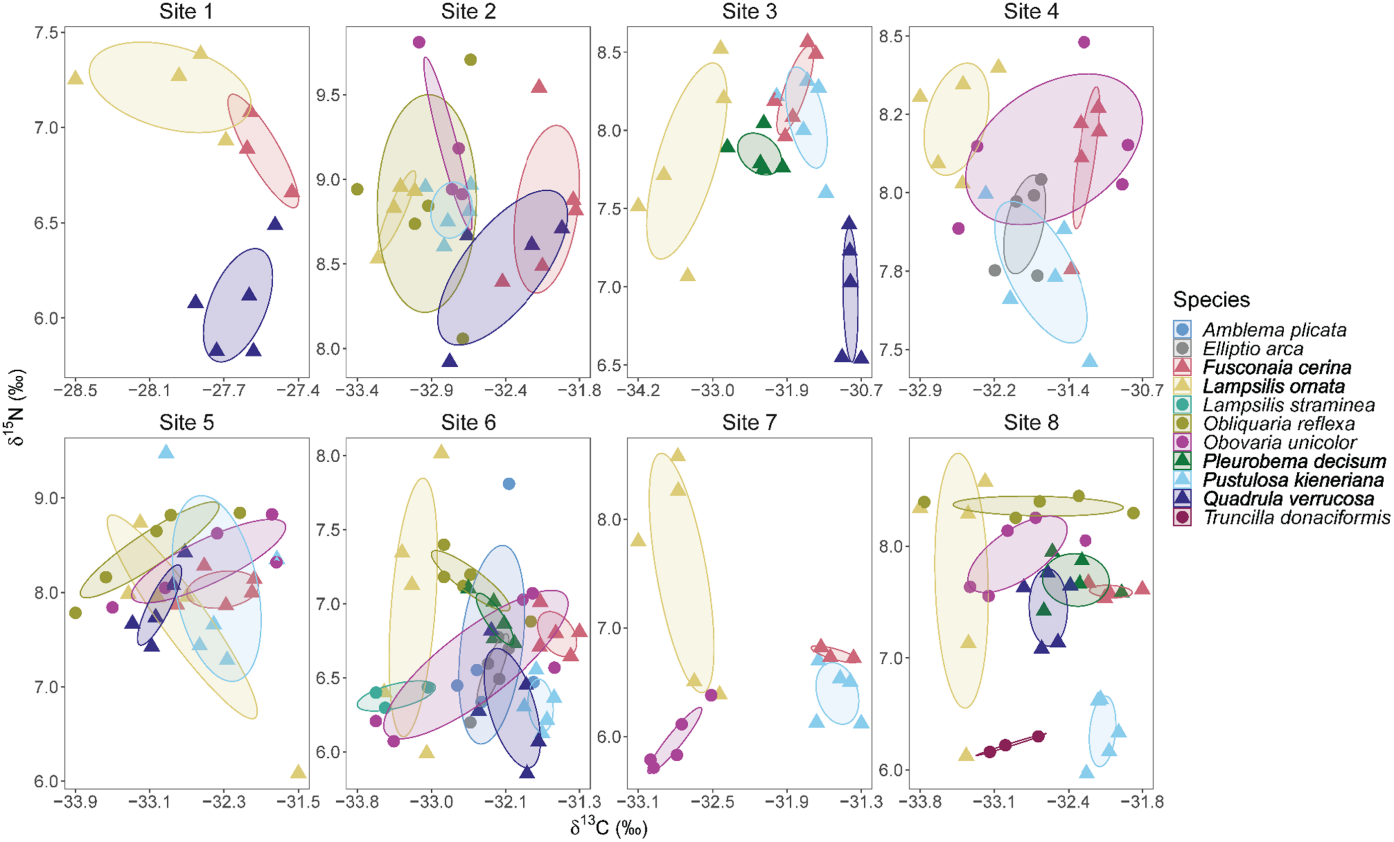
Isotope biplots and Bayesian standard ellipse areas (95% confidence intervals) of 11 mussel species from Site 1 (upstream) to Site 8 (downstream). Bolded names on legend with triangular shaped points are species with corresponding gill morphology data. Non‐bolded names on legend with circular shaped points do not have gill morphological data. Scales of *x*‐ and *y*‐axes differ.

Mussel trophic niche areas (SEA_B_) varied within and among species across sites (Table S1). Across sites and species, trophic niche area ranged from 0.03‰^2^ to 1.86‰^2^ with *Fusconaia cerina* from both Sites 7 and 8 having the smallest observed niche area, while *Lampsilis ornata* from Site 5 had the largest niche area. Within each site, the trophic niches of mussel species generally clustered in isotopic space regardless of species identity (Figure S2). However, despite clustering, we observed a low mean proportion of niche area overlap between co‐occurring species ranging from 0.04 to 0.18 across all eight sites (H2; Figure 3; Tables S2 and S3). While we observed some variability, site did not influence mussel trophic niche area (*H*_7_ = 8.67, *p* > 0.05), suggesting niche size consistency across populations. Conversely, species identity did influence the trophic niche area of a species (H1; *H*_10_ = 18.34; *p* < 0.05).

**FIGURE 4:**
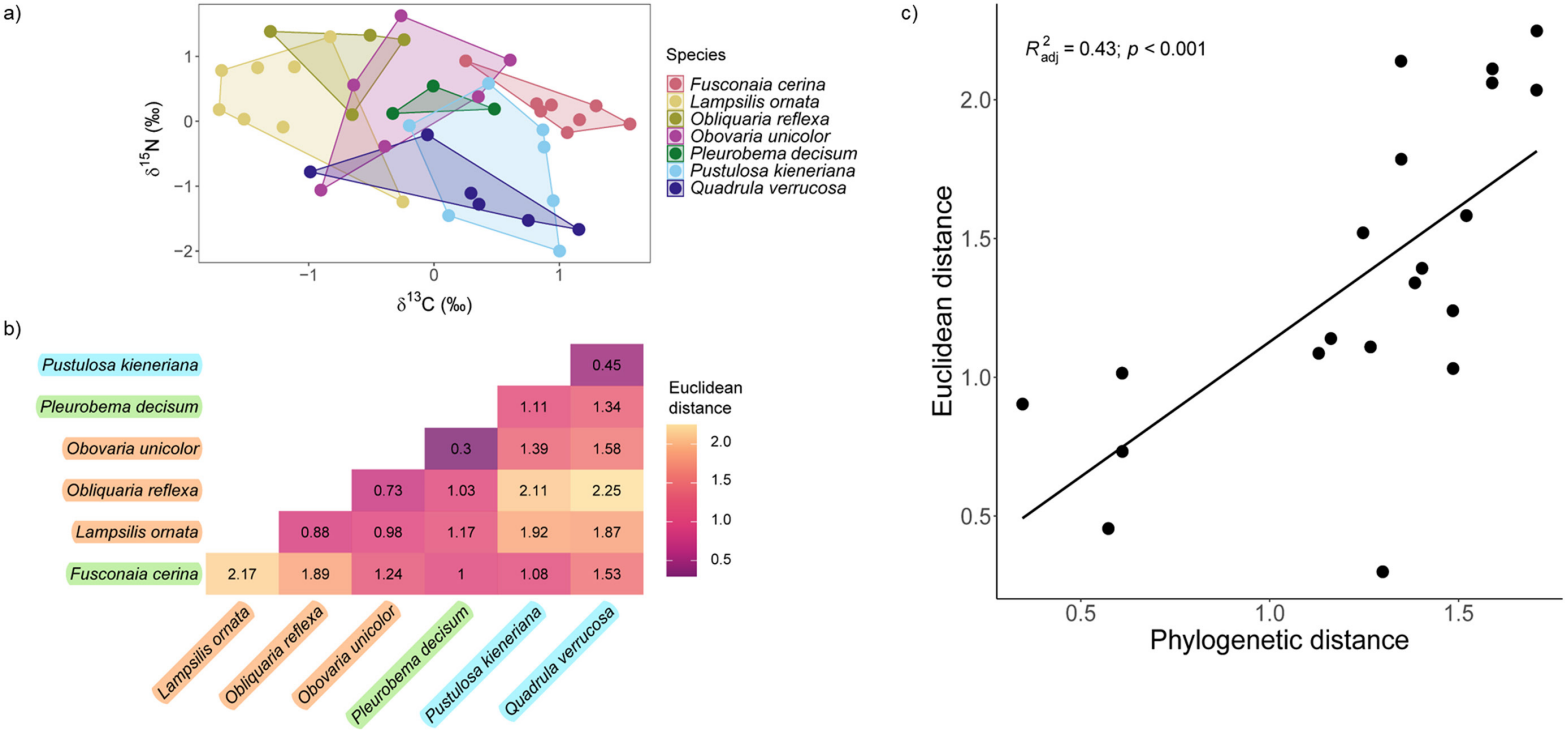
(a) Standardized stable isotopic signatures for seven of the 11 species (*n* ≥ 3 sites). Minimum convex hulls encompass all data points for each species across sites and represent their trophic niche area; (b) Euclidean distances between centroids of convex hulls (panel a) for each species pair; (c) Regression describing relationship between pairwise phylogenetic distances and Euclidean distances. Colors correspond to phylogenetic tribes: Lampsilini (orange), Pleurobemini (green), and Quadrulini (Blue).

PERMANOVA revealed an influence of species identity on mussel trophic dissimilarity (i.e., convex hulls; *F*_6,36_ = 9.27; *p* < 0.001; Figure 4a). Post hoc pairwise PERMANOVA confirmed isotopic differences between specific species pairs (Table S4). Isotopic dispersion did not differ among species (ANOVA: *F*_6,36_ = 1.01; *p* > 0.05), suggesting that variation in the central tendency of each species' convex hulls, rather than the overall spread, underlies trophic dissimilarity patterns. Pairwise phylogenetic distances and Euclidean distances in isotopic space of species pairs were positively related (*F*_1,18_ = 15.51; *R* ^2^ = 0.43; *p* < 0.001; H3, Figure 4b,c).

### 3.2 Gill Morphology and Resource Assimilation

**FIGURE 5:**
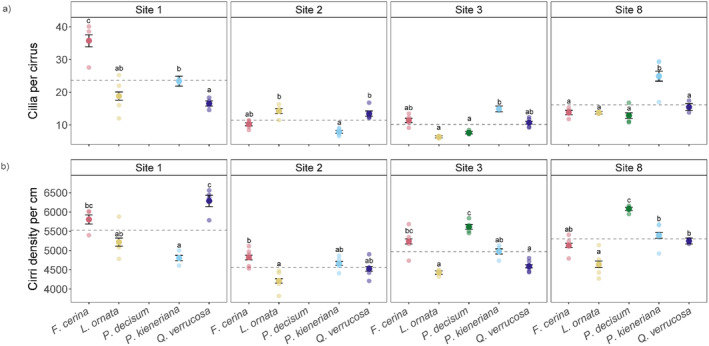
The number of cilia per cirrus (a) and latero‐frontal cirri density per cm of gill filament (b) for five mussel species *Fusconaia cerina* (red), *Lampsilis ornata* (yellow), *Pleurobema decisum* (green), *Pustulosa kieneriana* (blue), and *Quadrula verrucosa* (purple) from Sites 1, 2, 3 and 8. Large colored points are the average of the gill trait (±standard error) for each species. Lighter colored points correspond to the average of gill trait subsamples within an individual specimen. Dashed gray horizontal lines represent the average of the gill trait across all species within each site. Different letters indicate significant pairwise differences across species within each site.

There was intraspecific and interspecific variation in the gill anatomy of mussel species within and across our sites (Figure 5a,b). Across sites and species, the average number of CC ranged from 6.3 ± 0.3 to 35.7 ± 1.8, with *L. ornata* from Site 3 having the lowest observed CC on average, while *F. cerina* from Site 1 had the highest average number of CC (±SE; Table 1). In addition, across sites and species, the number of cirri per cm of gill filament (CD) ranged from 4210.3 ± 56.9 to 6287.8 ± 148.1, with *L. ornata* from Site 2 having the lowest CD levels on average, while *Q. verrucosa* from Site 1 had the highest average CD (±SE; Table 1).

With *P. decisum* excluded from analysis, there was an interactive effect between site and species identity on both the number of CC (*F*_8,45_ = 15.57; *p* < 0.0001; Figure 5a) and CD (*F*_8,45_ = 2.23; *p* < 0.05) (Figure 5b). When we included *P. decisum* with only Sites 3 and 8, there was also an interactive effect of site and species identity on both the number of CC (*F*_4,30_ = 2.97; *p* < 0.05; Figure 5a) and CD (*F*_4,30_ = 2.82; *p* < 0.05; Figure 5b). Moreover, we observed pairwise differences in the average CC and CD across both data subsets (Table S5).

**FIGURE 6:**
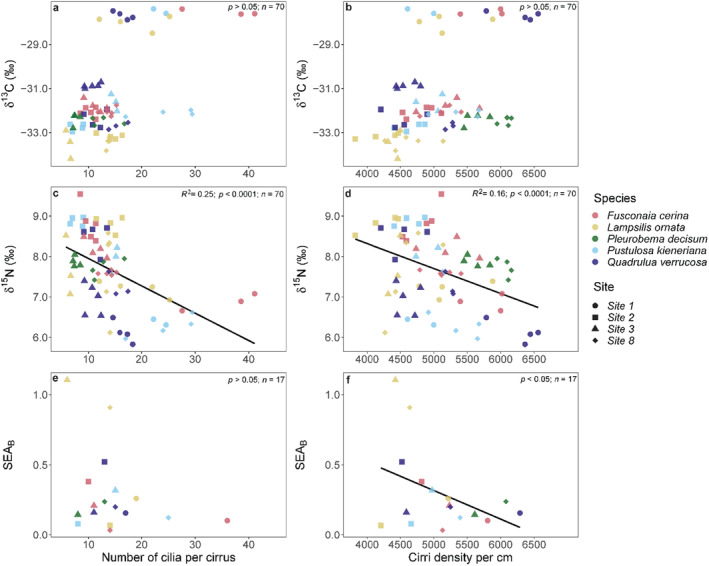
Relationship between the number of cilia per latero‐frontal cirrus (CC) and latero‐frontal cirri density per cm of gill filament (CD) with isotopic metrics across five mussel species from four sites: (a, b) Linear regression describing the relationship between δ^13^C with CC and CD, respectively; (c, d) Linear regression describing the relationship between δ^15^N and CC and CD, respectively; (e, f) Non‐parametric median‐based robust regression describing the relationship between mussel Bayesian standard ellipse area (SEA_B_) with CC and CD, respectively.

We evaluated the number of CC and CD as morphological traits that may affect the trophic niches of mussel species. Across sites and species, CC (*F*_1,68_ = 32.05; *p* < 0.0001; *R* ^2^ = 0.31) and CD values (*F*_1,68_ = 17.42; *p* < 0.0001; *R* ^2^ = 0.19) were positively related to mussel δ^13^C signatures. However, δ^13^C values at Site 1 were notably higher than at Sites 2 to 8, and we repeated this analysis without Site 1 and found no relationship between the number of CC (*F*_1,55_ = 0.273; *p* > 0.05; *R* ^2^ = 0; Figure 6a) or CD with δ^13^C (*F*_1,55_ = 2.44; *p* > 0.05; *R* ^2^ = 0.025; Figure 6b). Both the number of CC (*F*_1,68_ = 24.23; *p* < 0.0001; *R* ^2^ = 0.252; Figure 6c) and CD (*F*_1,68_ = 14.46; *p* < 0.001; *R* ^2^ = 0.16; Figure 6d) were negatively related to δ^15^N. We found that the number of CC (*V*_15_ = 48.00; *p* > 0.05; Figure 6e) was not negatively related to trophic niche area (i.e., SEA_B_), whereas CD was (H4; *V*_15_ = 17.00; *p* < 0.01; Figure 6f).

## 4 Discussion

Studies demonstrating how functional traits influence niche partitioning across communities are crucial to better understand the forces shaping community assembly. In the present study, we highlight the concordance between gill morphology and trophic niche area in dense and diverse assemblages of freshwater mussels that share feeding behaviors. We emphasize the influence of species identity and evolutionary history on the trophic niche, as trophic dissimilarity among species increased with phylogenetic distance. Our results suggest that within‐guild functional trait diversity is important in governing the niches of closely related, co‐occurring mussel species and may promote resource partitioning (Atkinson, van Ee, and Pfeiffer 2020; Haag 2012; Weingarten, Atkinson, and Jackson 2019) supporting the idea that evolutionary history affects resource assimilation (see Atkinson, van Ee, and Pfeiffer 2020). Specifically, we show that higher CD levels correspond to narrower niche areas and that although gill morphology varied among populations, the niche area of a species remained relatively consistent across sites.

### 4.1 Resource Assimilation is Shaped by Site and Species Identity

Location in the river network (site) and species identity influenced mussel resource assimilation. The diets of aquatic consumers can vary across habitat and water physiochemistry (Cochran‐Biederman and Winemiller 2010; Townsend and Hildrew 1994). In rivers, resource quantity and quality vary with location in the network and flow dynamics (Atkinson et al. 2019; Vannote et al. 1980). This variability likely resulted in isotopic differences of resources available to mussels. However, because we did not collect water resource samples from our study sites, we note that we can only make inferences regarding the specific types of food items a species may have assimilated.

Mussel isotopic signatures varied the most due to site identity, highlighting the role of environmental context in shaping food‐resource assimilation (Figure 1). Species upstream (Site 1) had notably enriched δ^13^C compared to those downstream (Sites 2 to 8). Based on δ^13^C, bacteria, fungi and soil subsidies may be important food sources in upstream reaches, while species downstream may rely more on phytoplankton, zooplankton, and riparian plant matter (Christian et al. 2004; Weber, Bauer, and Watters 2017). Mussel δ^13^C reflecting plant matter (Sites 2 to 8) may be due to the Sipsey wetland complex, which can increase concentrations of terrestrial organic matter at mid‐to‐downstream sites (Atkinson et al. 2019). Previous work suggested plant material was an important dietary item to freshwater mussels and our data (δ^13^C) also support this (Fogelman et al. 2022). There was no discernable pattern associated with mussel δ^15^N across sites, except that the average δ^15^N values were highest at Site 2 adjacent to the wetland complex. Wetlands foster bacterial production and anaerobic biogeochemical pathways such as denitrification (Czuba et al. 2018). The Sipsey wetland complex also retains inorganic N during periods of floodplain inundation (Atkinson et al. 2019) and elevated δ^15^N is indicative of material that has been colonized by microorganisms (Goedkoop, Akerblom, and Demandt 2006). Thus, the higher average δ^15^N values observed across mussel species at Site 2 may be attributable to increased availability and resultant assimilation of bacteria by mussels (Christian et al. 2004; Nichols and Garling 2000). Alternatively, the observed higher δ^15^N values at Site 2 may be due to the preferential reduction of ^14^NO_3_^−^ versus ^15^NO_3_^−^ by bacterial denitrifiers, which in turn, would result in a greater accumulation of ^15^N in the environment, including potential food sources (Mariotti, Germon, and Leclerc 1981; Mariotti et al. 1982; Peipoch, Martí, and Gacia 2012). Further research is needed to investigate the importance of wetland‐derived resources to instream consumers to better understand how they shape consumer resource assimilation in these ecosystems.

While species identity influenced resource assimilation, our results suggest that the trophic niche area (i.e., SEA_B_) of a species remained relatively consistent across different sites, indicating that our results were partially inconsistent with our first hypothesis (H1; Figure 1). Trophic niche consistency across environmental contexts has been documented in other taxa (Conti‐Jerpe, Pawlik, and Finelli 2022; Lu et al. 2022). This suggests that certain species can selectively feed to meet dietary requirements (Evan Ward and Shumway 2004), which might explain the constrained niche areas observed among species in this study. Even after data standardization, mussel species' trophic niche areas remained relatively consistent while central tendencies among species generally differed. In particular, *F. cerina* exhibited more pairwise differences in its central tendency than any other species. Our results further supports that mussels may preferentially assimilate specific food particles (Atkinson et al. 2010).

The low proportion of SEA_B_ overlap between co‐occurring species supports hypothesis two (H2; Figure 1) and aligns with studies demonstrating resource partitioning among co‐occurring filter‐feeding species (Conti‐Jerpe, Pawlik, and Finelli 2022; Dubois et al. 2007; Sánchez González et al. 2023). While there was variation in the niche area of a species there was still relatively little overlap in resource assimilation between co‐occurring species in this single river system. This implies that that despite sharing similar feeding modes, species are using different components of the available resource pool or might occupy different microhabitats with varying food resources.

We found that trophic dissimilarity among species increases with phylogenetic distance (H3; Figure 1). Previous work has shown that mussel species with greater evolutionary divergence exhibit larger differences in their soft‐tissue stoichiometry (Atkinson, van Ee, and Pfeiffer 2020). Thus, in addition to shaping stoichiometric niches, evolutionary history likely influences resource selection, resulting in more closely related taxa assimilating more similar resources (Atkinson, van Ee, and Pfeiffer 2020; González et al. 2018). Resource stoichiometry has been found to influence the foraging behaviors of both terrestrial and aquatic animals (Rizzuto et al. 2021; Schatz and McCauley 2007). Therefore, mussels in the present study might be actively choosing food items to minimize stoichiometric mismatches between their tissue composition and the available resources. While additional research is warranted to better understand the interplay between phylogeny and stoichiometry in shaping resource assimilation, we suggest that these factors play a crucial role in shaping the trophic niches of species within the same functional feeding group.

### 4.2 Gill Morphology Influences the Trophic Niche and is Shaped by Location in the River Network

Mussels use their labial palps and gills to selectively capture food particles. Selectivity on the gills is largely driven by particle size, with increased clearance of smaller particles being linked to a higher density of latero‐frontal cirri (CD) and a greater number of CC associated with the gills (Galbraith et al. 2009; Silverman et al. 1997). Our findings support the hypothesis that gill morphology may constrain particle size passage and subsequent assimilation (Evan Ward and Shumway 2004). Specifically, we observed no relationship between CC and trophic niche area (H4; Figure 1), but both traits (CC and CD) tended to be higher at Site 1, which had more enriched δ^13^C, perhaps indicating higher assimilation of smaller particles such as bacteria (Weber, Bauer, and Watters 2017). Moreover, we found a negative relationship between CD and trophic niche area (H4; Figure 1), suggesting that higher densities may constrain the range of particle size classes a mussel species can assimilate, thus narrowing their trophic niche area (H4; Figure 1). Similar relationships between morphology and resource use based on food item size have also been observed in other filter‐feeders (Castillo‐Rivera, Kobelkowsky, and Zamayoa 1996) and other taxa (e.g., rodents and amphipods; Brown and Liberman 1973; Premate et al. 2021).

Our results show that morphological traits can differ among spatially separated populations within a single system (H1; Figure 1). The observed differences in the number of CC and CD may be due to variation in abiotic factors (e.g., turbidity; Tuttle‐Raycraft and Ackerman 2019), or in biotic factors not related to particle capture (e.g., respiration or glochidia brooding), or in the food resources available to these animals across sites (Atkinson et al. 2019). Changes in the relative size of feeding structures (i.e., gills and labial palps) in response to particle composition have been reported in both marine and freshwater bivalves (Capelle et al. 2021; Compton et al. 2008; Payne et al. 1995). Therefore, population differences in CC and CD may reflect a phenotypic response to potential variation in resources across our sites. Whether these differences are due to phenotypic plasticity or other factors such as species interactions or historical contingencies warrants further study.

While CD was negatively related to trophic niche area, the observed consistency of niche dimensions suggests that other mechanisms are likely to be constraining. For instance, Ward et al. (1998) found that experimental deactivation of the latero‐frontal cirri resulted in a reduction but not complete cessation of particle capture. In addition, Brillant and MacDonald (2002) observed that following ingestion, marine bivalves can differentiate between chemically distinct but morphologically equivalent particles. Other selection mechanisms have been documented in filter‐feeders (Evan Ward and Shumway 2004; Rubenstein and Koehl 1977). Future work is needed to better understand the factors that impact feeding morphology, particle selectivity, and the trophic niches of filter‐feeding animals.

## 5 Conclusions

The results of our study suggest that both phylogenetic relatedness and interspecific differences in morphology may promote trophic niche partitioning among species, while intraspecific differences in gill morphology across populations may be a phenotypic response that constrains the trophic niche under potentially variable resource regimes. Therefore, the evolutionary history of a species combined with intraguild variation in feeding morphology are likely key factors underlying trophic assimilation patterns and species coexistence in dense and species‐rich communities. Our findings also support the hypothesis that CD is important for particle selectivity and broaden our understanding of filter‐feeding by demonstrating a relationship between CD and trophic niche area. Further research should examine whether similar patterns occur across different river systems and species. Moreover, additional work is needed to determine the influence of other functional traits and microhabitat selection on trophic niche occupancy among members of diverse communities that share feeding modes, as this will help us to better understand the mechanisms underlying the coexistence of ecologically similar species.

## Author Contributions

**Matthew B. Lodato:** conceptualization (equal), data curation (lead), formal analysis (lead), investigation (supporting), methodology (supporting), visualization (lead), writing – original draft (lead), writing – review and editing (lead). **Brian C. van Ee:** conceptualization (equal), data curation (supporting), investigation (lead), methodology (lead), writing – review and editing (supporting). **Carla L. Atkinson:** conceptualization (equal), data curation (supporting), formal analysis (supporting), funding acquisition (lead), investigation (lead), methodology (lead), visualization (supporting), writing – original draft (supporting), writing – review and editing (supporting).

## Conflicts of Interest

The authors declare no conflicts of interest.

## Supporting information


Appendix S1

## Data Availability

Data can be found at the Open Science Framework at https://osf.io/wrhd7/?view_only=411162fbc3db4523ab795077948cb87e.
