# Supplementary material for: Linking Phylogeny and Morphology to Resource Assimilation Within Aquatic Assemblages
Source: Ecol Evol. 2024 Nov 24;14(11):e70641. doi: 10.1002/ece3.70641 (PMC11586237; doi:10.1002/ece3.70641)
Supplement: Supplementary file 1 — Appendix S1 [file ECE3-14-e70641-s001.docx]

S1: We collected live adult mussels (Family Unionidae) from eight sites along the Sipsey River, Alabama, USA, between August and October 2016. All sampling was conducted during baseflow conditions using both visual and tactile techniques. At the time of collection, we did not determine the age, sex, or reproductive status of individuals because most mussels are not sexually dimorphic and must be examined under a microscope to determine these factors. However, we assume our sampling integrates some variability in these factors. During each sampling event, mussels were temporarily held in submerged mesh bags placed in flowing sections of the river. Following a sampling event, all mussels were transported on ice to the lab for further processing.

# **Table S1**: Mean body lengths and isotopic-metrics ± standard error (SE) for 11 mussel species across eight sites in the Sipsey River, Alabama. Isotopic-metrics include normalized mussel soft-tissue δ^13^C and δ^15^N measurements, and corresponding trophic niche area (Bayesian standard ellipse area; SEA_B_) estimates. *N* is the number of samples per species within each site. NA indicates no corresponding value is associated with variable.

| **Site** | **Species** | **Phylogenetic tribe** | **Length (mm) ± SE** | **Mean δ^13^C ± SE** | **Mean δ^15^N ± SE** | **SEAB** | ***N*** |  |
| --- | --- | --- | --- | --- | --- | --- | --- | --- |
|  |  |  |  |  |  |  |  |  |
| Site 1 | *Fusconaia cerina* | Pleurobemini | 52.57 (4.38) | -27.54 (0.07) | 6.88 (0.12) | 0.10 | 3 |  |
|  | *Lampsilis ornata* | Lampsilini | 86.23 (3.38) | -28.01 (0.17) | 7.21 (0.1) | 0.26 | 4 |  |
|  | *Pustulosa kieneriana* | Quadrulini | 50.25 (12.05) | -27.49 (0.10) | 6.38 0.07) | NA | 2 |  |
|  | *Quadrula verrucosa* | Quadrulini | 68.16 (7.48) | -27.67 (0.07) | 6.07 (0.12) | 0.16 | 5 |  |
| Site 2 | *Fusconaia cerina* | Pleurobemini | 54.70 (1.99) | -32.07 (0.10) | 8.82 (0.20) | 0.38 | 5 |  |
|  | *Lampsilis ornata* | Lampsilini | 104.83 (4.74) | -33.16 (0.06) | 8.81 (0.1) | 0.07 | 4 |  |
|  | *Obliquaria reflexa* | Lampsilini | 48.66 (4.74) | -32.94 (0.15) | 8.86 (0.26) | 0.76 | 5 |  |
|  | *Obovaria unicolor* | Lampsilini | 44.40 (2.16) | -32.79 (0.07) | 9.21 (0.21) | 0.18 | 4 |  |
|  | *Pustulosa kieneriana* | Quadrulini | 51.60 (2.22) | -32.76 (0.06) | 8.82 (0.07) | 0.08 | 5 |  |
|  | *Quadrula verrucosa* | Quadrulini | 92.05 (19.73) | -32.39 (0.19) | 8.48 (0.19) | 0.52 | 4 |  |
| Site 3 | *Fusconaia cerina* | Pleurobemini | 44.60 (3.53) | -31.75 (0.12) | 8.26 (0.12) | 0.21 | 5 |  |
|  | *Lampsilis ornata* | Lampsilini | 71.06 (11.27) | -33.44 (0.26) | 7.80 (0.26) | 1.10 | 5 |  |
|  | *Pleurobema decisum* | Pleurobemini | 49.36 (4.20) | -32.30 (0.14) | 7.85 (0.05) | 0.15 | 5 |  |
|  | *Pustulosa kieneriana* | Quadrulini | 47.08 (1.85) | -31.57 (0.13) | 8.08 (0.13) | 0.32 | 5 |  |
|  | *Quadrula verrucosa* | Quadrulini | NA | -30.88 (0.05) | 6.95 (0.18) | 0.16 | 5 |  |
| Site 4 | *Elliptio arca* | Pleurobemini | 69.00 (3.20) | -31.87 (0.09) | 7.90 (0.06) | 0.10 | 5 |  |
|  | *Fusconaia cerina* | Pleurobemini | 48.10 (3.11) | -31.24 (0.06) | 8.11 (0.09) | 0.08 | 5 |  |
|  | *Lampsilis ornata* | Lampsilini | 90.22 (2.42) | -32.57 (0.14) | 8.23 (0.07) | 0.19 | 5 |  |
|  | *Obovaria unicolor* | Lampsilini | 34.82 (3.76) | -31.57 (0.37) | 8.14 (0.10) | 0.69 | 5 |  |
|  | *Pustulosa kieneriana* | Quadrulini | 43.98 (3.45) | -31.70 (0.19) | 7.75 (0.09) | 0.30 | 5 |  |
| Site 5 | *Fusconaia cerina* | Pleurobemini | 48.76 (2.53) | -32.32 (0.15) | 8.03 (0.08) | 0.24 | 5 |  |
|  | *Lampsilis ornata* | Lampsilini | 82.82 (5.97) | -32.75 (0.32) | 7.74 (0.44) | 1.86 | 5 |  |
|  | *Obliquaria reflexa* | Lampsilini | 40.12 (1.17) | -33.09(0.30) | 8.45 (0.21) | 0.82 | 5 |  |
|  | *Obovaria unicolor* | Lampsilini | 37.40 (2.43) | -32.47 (0.33) | 8.33 (0.18) | 0.90 | 5 |  |
|  | *Pustulosa kieneriana* | Quadrulini | 43.10 (5.01) | -32.39 (0.19) | 8.04 (0.40) | 1.47 | 5 |  |
|  | *Quadrula verrucosa* | Quadrulini | 80.36 (9.90) | -32.99 (0.09) | 7.87 (0.17) | 0.24 | 5 |  |
| Site 6 | *Amblema plicata* | Amblemini | 69.98 (7.62) | -32.30 (0.15) | 6.72 (0.27) | 0.80 | 5 |  |
|  | *Elliptio arca* | Pleurobemini | 64.34 (4.52) | -32.29 (0.07) | 6.55 (0.10) | 0.11 | 5 |  |
|  | *Fusconaia cerina* | Pleurobemini | 48.30 (2.75) | -31.55 (0.09) | 6.80 (0.06) | 0.10 | 5 |  |
|  | *Lampsilis ornata* | Lampsilini | 85.52 (8.68) | -33.19 (0.11) | 6.98 (0.36) | 0.75 | 5 |  |
|  | *Lampsilis straminea* | Lampsilini | 42.50 (6.17) | -33.38 (0.18) | 6.38 (0.04) | 0.10 | 3 |  |
|  | *Obliquaria reflexa* | Lampsilini | 46.46 (4.00) | -32.54 (0.18) | 7.16 (0.08) | 0.21 | 5 |  |
|  | *Obovaria unicolor* | Lampsilini | 35.32 (3.14) | -32.48 (0.43) | 6.59 (0.20) | 1.29 | 5 |  |
|  | *Pleurobema decisum* | Pleurobemini | 53.50 (3.57) | -32.26 (0.09) | 6.90 (0.07) | 0.09 | 5 |  |
|  | *Pustulosa kieneriana* | Quadrulini | 48.64 (2.17) | -31.74 (0.06) | 6.31 (0.07) | 0.08 | 5 |  |
|  | *Quadrula verrucosa* | Quadrulini | 84.64 (2.17) | -32.07 (0.13) | 6.29 (0.17) | 0.38 | 5 |  |
| Site 7 | *Fusconaia cerina* | Pleurobemini | 43.60 (3.12) | -31.53 (0.08) | 6.76 (0.03) | 0.03 | 3 |  |
|  | *Lampsilis ornata* | Lampsilini | 79.06 (5.29) | -32.73 (0.10) | 7.51 (0.45) | 0.79 | 5 |  |
|  | *Obovaria unicolor* | Lampsilini | 34.98 (2.56) | -32.78(0.09) | 5.96 (0.12) | 0.14 | 5 |  |
|  | *Pustulosa kieneriana* | Quadrulini | 43.75 (3.80) | -31.51 (0.07) | 6.40 (0.12) | 0.15 | 5 |  |
| Site 8 | *Fusconaia cerina* | Pleurobemini | 36.74 (2.60) | -32.05 (0.08) | 7.60 (0.02) | 0.03 | 5 |  |
|  | *Lampsilis ornata* | Lampsilini | 79.58 (8.26) | -33.44 (0.10) | 7.69(0.47) | 0.91 | 5 |  |
|  | *Lampsilis straminea* | Lampsilini | 74.10 (NA) | -33.16 (NA) | 7.14 (NA) | NA | 1 |  |
|  | *Obliquaria reflexa* | Lampsilini | 50.14 (6.08) | -32.73 (0.32) | 8.36 (0.04) | 0.23 | 5 |  |
|  | *Obovaria unicolor* | Lampsilini | 41.78 (2.50) | -32.92 (0.19) | 7.93 (0.14) | 0.44 | 5 |  |
|  | *Pleurobema decisum* | Pleurobemini | 46.32 (3.40) | -32.38 (0.13) | 7.70(0.10) | 0.24 | 5 |  |
|  | *Pustulosa kieneriana* | Quadrulini | 41.82 (4.68) | -32.13 (0.05) | 6.34 (0.13) | 0.12 | 5 |  |
|  | *Quadrula verrucosa* | Quadrulini | 71.88 (7.33) | -32.64 (0.07) | 7.45 (0.04) | 0.20 | 5 |  |
|  | *Truncilla donaciformis* | Lampsilini | 23.93 (2.73) | -32.98 (0.13) | 6.23 (0.14) | 0.06 | 3 |  |

# **Table S2**: The proportion of trophic niche area (Bayesian standard ellipse area) overlap between co-occurring species within each site.

| **Site** | **Species A** | **Species B** | **Proportion of niche area overlap** |  |
| --- | --- | --- | --- | --- |
|  |  |  |  |  |
| Site 1 | *Fusconaia cerina* | *Lampsilis ornata* | 0.19 |  |
|  | *Fusconaia cerina* | *Quadrula verrucosa* | 0.14 |  |
|  | *Lampsilis ornata* | *Quadrula verrucosa* | 0.03 |  |
| Site 2 | *Pustulosa kieneriana* | *Fusconaia cerina* | 0.06 |  |
|  | *Pustulosa kieneriana* | *Lampsilis ornata* | 0.11 |  |
|  | *Pustulosa kieneriana* | *Obliquaria reflexa* | 0.11 |  |
|  | *Pustulosa kieneriana* | *Obovaria unicolor* | 0.24 |  |
|  | *Pustulosa kieneriana* | *Quadrula verrucosa* | 0.12 |  |
|  | *Fusconaia cerina* | *Lampsilis ornata* | < 0.0001 |  |
|  | *Fusconaia cerina* | *Obliquaria reflexa* | 0.18 |  |
|  | *Fusconaia cerina* | *Obovaria unicolor* | 0.07 |  |
|  | *Fusconaia cerina* | *Quadrula verrucosa* | 0.47 |  |
|  | *Lampsilis ornata* | *Obliquaria reflexa* | 0.06 |  |
|  | *Lampsilis ornata* | *Obovaria unicolor* | 0.03 |  |
|  | *Lampsilis ornata* | *Quadrula verrucosa* | 0.003 |  |
|  | *Obliquaria reflexa* | *Obovaria unicolor* | 0.13 |  |
|  | *Obliquaria reflexa* | *Quadrula verrucosa* | 0.35 |  |
|  | *Obovaria unicolor* | *Quadrula verrucosa* | 0.08 |  |
| Site 3 | *Pustulosa kieneriana* | *Fusconaia cerina* | 0.34 |  |
|  | *Pustulosa kieneriana* | *Lampsilis ornata* | 0.05 |  |
|  | *Pustulosa kieneriana* | *Pleurobema decisum* | 0.14 |  |
|  | *Pustulosa kieneriana* | *Quadrula verrucosa* | 0.10 |  |
|  | *Fusconaia cerina* | *Lampsilis ornata* | 0.03 |  |
|  | *Fusconaia cerina* | *Pleurobema decisum* | 0.23 |  |
|  | *Fusconaia cerina* | *Quadrula verrucosa* | < 0.0001 |  |
|  | *Lampsilis ornata* | *Pleurobema decisum* | 0.08 |  |
|  | *Lampsilis ornata* | *Quadrula verrucosa* | < 0.0001 |  |
|  | *Pleurobema decisum* | *Quadrula verrucosa* | < 0.0001 |  |
| Site 4 | *Pustulosa kieneriana* | *Elliptio arca* | 0.32 |  |
|  | *Pustulosa kieneriana* | *Fusconaia cerina* | 0.09 |  |
|  | *Pustulosa kieneriana* | *Lampsilis ornata* | 0.17 |  |
|  | *Pustulosa kieneriana* | *Obovaria unicolor* | 0.24 |  |
|  | *Elliptio arca* | *Fusconaia cerina* | 0.07 |  |
|  | *Elliptio arca* | *Lampsilis ornata* | 0.06 |  |
|  | *Elliptio arca* | *Obovaria unicolor* | 0.15 |  |
|  | *Fusconaia cerina* | *Lampsilis ornata* | < 0.0001 |  |
|  | *Fusconaia cerina* | *Obovaria unicolor* | 0.10 |  |
|  | *Lampsilis ornata* | *Obovaria unicolor* | 0.10 |  |
| Site 5 | *Pustulosa kieneriana* | *Fusconaia cerina* | 0.17 |  |
|  | *Pustulosa kieneriana* | *Lampsilis ornata* | 0.36 |  |
|  | *Pustulosa kieneriana* | *Obliquaria reflexa* | 0.18 |  |
|  | *Pustulosa kieneriana* | *Obovaria unicolor* | 0.24 |  |
|  | *Pustulosa kieneriana* | *Quadrula verrucosa* | 0.11 |  |
|  | *Fusconaia cerina* | *Lampsilis ornata* | 0.12 |  |
|  | *Fusconaia cerina* | *Obliquaria reflexa* | 0.06 |  |
|  | *Fusconaia cerina* | *Obovaria unicolor* | 0.25 |  |
|  | *Fusconaia cerina* | *Quadrula verrucosa* | 0.22 |  |
|  | *Lampsilis ornata* | *Obliquaria reflexa* | 0.12 |  |
|  | *Lampsilis ornata* | *Obovaria unicolor* | 0.15 |  |
|  | *Lampsilis ornata* | *Quadrula verrucosa* | 0.11 |  |
|  | *Obliquaria reflexa* | *Obovaria unicolor* | 0.34 |  |
|  | *Obliquaria reflexa* | *Quadrula verrucosa* | 0.12 |  |
|  | *Obovaria unicolor* | *Quadrula verrucosa* | 0.19 |  |
| Site 6 | *Amblema plicata* | *Pustulosa kieneriana* | 0.09 |  |
|  | *Amblema plicata* | *Elliptio arca* | 0.11 |  |
|  | *Amblema plicata* | *Fusconaia cerina* | 0.09 |  |
|  | *Amblema plicata* | *Lampsilis ornata* | 0.16 |  |
|  | *Amblema plicata* | *Lampsilis straminea* | 0.05 |  |
|  | *Amblema plicata* | *Obliquaria reflexa* | 0.13 |  |
|  | *Amblema plicata* | *Obovaria unicolor* | 0.31 |  |
|  | *Amblema plicata* | *Pleurobema decisum* | 0.09 |  |
|  | *Amblema plicata* | *Quadrula verrucosa* | 0.34 |  |
|  | *Pustulosa kieneriana* | *Elliptio arca* | 0.01 |  |
|  | *Pustulosa kieneriana* | *Fusconaia cerina* | 0.11 |  |
|  | *Pustulosa kieneriana* | *Lampsilis ornata* | < 0.0001 |  |
|  | *Pustulosa kieneriana* | *Lampsilis straminea* | < 0.0001 |  |
|  | *Pustulosa kieneriana* | *Obliquaria reflexa* | 0.03 |  |
|  | *Pustulosa kieneriana* | *Obovaria unicolor* | 0.05 |  |
|  | *Pustulosa kieneriana* | *Pleurobema decisum* | 0.09 |  |
|  | *Pustulosa kieneriana* | *Quadrula verrucosa* | 0.22 |  |
|  | *Elliptio arca* | *Fusconaia cerina* | 0.10 |  |
|  | *Elliptio arca* | *Lampsilis ornata* | 0.00 |  |
|  | *Elliptio arca* | *Lampsilis straminea* | 0.05 |  |
|  | *Elliptio arca* | *Obliquaria reflexa* | 0.11 |  |
|  | *Elliptio arca* | *Obovaria unicolor* | 0.08 |  |
|  | *Elliptio arca* | *Pleurobema decisum* | 0.19 |  |
|  | *Elliptio arca* | *Quadrula verrucosa* | 0.20 |  |
|  | *Fusconaia cerina* | *Lampsilis ornata* | < 0.0001 |  |
|  | *Fusconaia cerina* | *Lampsilis straminea* | < 0.0001 |  |
|  | *Fusconaia cerina* | *Obliquaria reflexa* | 0.09 |  |
|  | *Fusconaia cerina* | *Obovaria unicolor* | 0.11 |  |
|  | *Fusconaia cerina* | *Pleurobema decisum* | < 0.0001 |  |
|  | *Fusconaia cerina* | *Quadrula verrucosa* | 0.05 |  |
|  | *Lampsilis ornata* | *Lampsilis straminea* | 0.1 |  |
|  | *Lampsilis ornata* | *Obliquaria reflexa* | 0.09 |  |
|  | *Lampsilis ornata* | *Obovaria unicolor* | 0.19 |  |
|  | *Lampsilis ornata* | *Pleurobema decisum* | 0.02 |  |
|  | *Lampsilis ornata* | *Quadrula verrucosa* | 0.04 |  |
|  | *Lampsilis straminea* | *Obliquaria reflexa* | 0 |  |
|  | *Lampsilis straminea* | *Obovaria unicolor* | 0.11 |  |
|  | *Lampsilis straminea* | *Pleurobema decisum* | < 0.0001 |  |
|  | *Lampsilis straminea* | *Quadrula verrucosa* | 0.05 |  |
|  | *Obliquaria reflexa* | *Obovaria unicolor* | 0.08 |  |
|  | *Obliquaria reflexa* | *Pleurobema decisum* | 0.28 |  |
|  | *Obliquaria reflexa* | *Quadrula verrucosa* | 0.16 |  |
|  | *Obovaria unicolor* | *Pleurobema decisum* | 0.07 |  |
|  | *Obovaria unicolor* | *Quadrula verrucosa* | 0.20 |  |
|  | *Pleurobema decisum* | *Quadrula verrucosa* | 0.18 |  |
| Site 7 | *Pustulosa kieneriana* | *Fusconaia cerina* | 0.16 |  |
|  | *Pustulosa kieneriana* | *Lampsilis ornata* | < 0.0001 |  |
|  | *Pustulosa kieneriana* | *Obovaria unicolor* | < 0.0001 |  |
|  | *Fusconaia cerina* | *Lampsilis ornata* | < 0.0001 |  |
|  | *Fusconaia cerina* | *Obovaria unicolor* | < 0.0001 |  |
|  | *Lampsilis ornata* | *Obovaria unicolor* | 0.06 |  |
| Site 8 | *Pustulosa kieneriana* | *Fusconaia cerina* | 0 |  |
|  | *Pustulosa kieneriana* | *Lampsilis ornata* | < 0.0001 |  |
|  | *Pustulosa kieneriana* | *Obliquaria reflexa* | 0 |  |
|  | *Pustulosa kieneriana* | *Obovaria unicolor* | < 0.0001 |  |
|  | *Pustulosa kieneriana* | *Pleurobema decisum* | 0.002 |  |
|  | *Pustulosa kieneriana* | *Truncilla donaciformis* | 0.009 |  |
|  | *Pustulosa kieneriana* | *Quadrula verrucosa* | 0.005 |  |
|  | *Fusconaia cerina* | *Lampsilis ornata* | < 0.0001 |  |
|  | *Fusconaia cerina* | *Obliquaria reflexa* | 0 |  |
|  | *Fusconaia cerina* | *Obovaria unicolor* | 0.01 |  |
|  | *Fusconaia cerina* | *Pleurobema decisum* | 0.14 |  |
|  | *Fusconaia cerina* | *Truncilla donaciformis* | 0 |  |
|  | *Fusconaia cerina* | *Quadrula verrucosa* | 0.05 |  |
|  | *Lampsilis ornata* | *Obliquaria reflexa* | 0.07 |  |
|  | *Lampsilis ornata* | *Obovaria unicolor* | 0.20 |  |
|  | *Lampsilis ornata* | *Pleurobema decisum* | 0.04 |  |
|  | *Lampsilis ornata* | *Truncilla donaciformis* | 0.01 |  |
|  | *Lampsilis ornata* | *Quadrula verrucosa* | 0.05 |  |
|  | *Obliquaria reflexa* | *Obovaria unicolor* | 0.20 |  |
|  | *Obliquaria reflexa* | *Pleurobema decisum* | 0.05 |  |
|  | *Obliquaria reflexa* | *Truncilla donaciformis* | 0 |  |
|  | *Obliquaria reflexa* | *Quadrula verrucosa* | 0.03 |  |
|  | *Obovaria unicolor* | *Pleurobema decisum* | 0.26 |  |
|  | *Obovaria unicolor* | *Truncilla donaciformis* | 0 |  |
|  | *Obovaria unicolor* | *Quadrula verrucosa* | 0.21 |  |
|  | *Pleurobema decisum* | *Truncilla donaciformis* | 0 |  |
|  | *Pleurobema decisum* | *Quadrula verrucosa* | 0.45 |  |
|  | *Truncilla donaciformis* | *Quadrula verrucosa* | 0 |  |

# **Table S3**: The mean proportion of trophic niche area (Bayesian standard ellipse area) overlap across species within each site.

| **Site** | **Proportion of niche area overlap** |  |
| --- | --- | --- |
|  |  |  |
| Site 1 | 0.12 |  |
| Site 2 | 0.13 |  |
| Site 3 | 0.10 |  |
| Site 4 | 0.13 |  |
| Site 5 | 0.18 |  |
| Site 6 | 0.10 |  |
| Site 7 | 0.04 |  |
| Site 8 | 0.06 |  |

# **Table S4:** Pairwise PERMANOVA post-hoc analysis on standardized isotopic signatures for seven of the 11 species. P-values adjusted using Bonferroni method. Bolded values indicate significance.

| **Species A** | **Species B** | ***F*-value** | **R^2^** | ***P*-value** |  |
| --- | --- | --- | --- | --- | --- |
|  |  |  |  |  |  |
| *Fusconaia cerina* | *Lampsilis ornata* | 32.45 | 0.70 | **0.006** |  |
| *Fusconaia cerina* | *Obliquaria reflexa* | 23.46 | 0.70 | **0.038** |  |
| *Fusconaia cerina* | *Obovaria unicolor* | 6.88 | 0.36 | **0.044** |  |
| *Fusconaia cerina* | *Pleurobema decisum* | 6.71 | 0.43 | 0.283 |  |
| *Fusconaia cerina* | *Pustulosa kieneriana* | 6.02 | 0.30 | 0.155 |  |
| *Fusconaia cerina* | *Quadrula verrucosa* | 15.46 | 0.56 | **0.008** |  |
| *Lampsilis ornata* | *Obliquaria reflexa* | 2.45 | 0.20 | 1.000 |  |
| *Lampsilis ornata* | *Obovaria unicolor* | 3.55 | 0.23 | 0.819 |  |
| *Lampsilis ornata* | *Pleurobema decisum* | 4.82 | 0.35 | 0.397 |  |
| *Lampsilis ornata* | *Pustulosa kieneriana* | 19.52 | 0.58 | **0.008** |  |
| *Lampsilis ornata* | *Quadrula verrucosa* | 16.50 | 0.58 | **0.033** |  |
| *Obliquaria reflexa* | *Obovaria unicolor* | 1.62 | 0.17 | 1.000 |  |
| *Obliquaria reflexa* | *Pleurobema decisum* | 4.19 | 0.46 | 1.000 |  |
| *Obliquaria reflexa* | *Pustulosa kieneriana* | 14.51 | 0.59 | **0.046** |  |
| *Obliquaria reflexa* | *Quadrula verrucosa* | 17.04 | 0.68 | 0.099 |  |
| *Obovaria unicolor* | *Pleurobema decisum* | 0.14 | 0.02 | 1.000 |  |
| *Obovaria unicolor* | *Pustulosa kieneriana* | 5.36 | 0.31 | 0.399 |  |
| *Obovaria unicolor* | *Quadrula verrucosa* | 6.46 | 0.39 | 0.222 |  |
| *Pleurobema decisum* | *Pustulosa kieneriana* | 3.40 | 0.27 | 1.000 |  |
| *Pleurobema decisum* | *Quadrula verrucosa* | 5.85 | 0.46 | 0.775 |  |
| *Pustulosa kieneriana* | *Quadrula verrucosa* | 1.39 | 0.10 | 1.000 |  |

**Table S5**: Results from Tukey’s HSD post-hoc analysis examining pairwise differences of average gill traits ± standard error (SE) between species within each site for subdivided data excluding *Pleurobema decisum* and data subset including this species. *P*-values adjusted using the Tukey method. Bold values indicate significance.

| **Morphological trait** | **Site** | **Species A** | **Species B** | **Estimated differences** | **SE** | **df** | ***P*-value** |  |
| --- | --- | --- | --- | --- | --- | --- | --- | --- |
|  |  |  |  |  |  |  |  |  |
| Cilia per cirri | Site 1 | *Fusconaia cerina* | *Lampsilis ornata* | 16.94 | 2.24 | 46 | **<0.0001** |  |
|  |  | *Fusconaia cerina* | *Pustulosa kieneriana* | 12.35 | 2.67 | 46 | **0.0002** |  |
|  |  | *Fusconaia cerina* | *Quadrula verrucosa* | 19.21 | 2.24 | 46 | **<0.0001** |  |
|  |  | *Lampsilis ornata* | *Pustulosa kieneriana* | -4.58 | 2.54 | 46 | 0.284 |  |
|  |  | *Lampsilis ornata* | *Quadrula verrucosa* | 2.28 | 2.07 | 46 | 0.692 |  |
|  |  | *Pustulosa kieneriana* | *Quadrula verrucosa* | 6.86 | 2.54 | 46 | **0.046** |  |
|  | Site 2 | *Fusconaia cerina* | *Lampsilis ornata* | -4.02 | 1.97 | 46 | 0.186 |  |
|  |  | *Fusconaia cerina* | *Pustulosa kieneriana* | 2.34 | 1.97 | 46 | 0.636 |  |
|  |  | *Fusconaia cerina* | *Quadrula verrucosa* | -3.24 | 1.97 | 46 | 0.361 |  |
|  |  | *Lampsilis ornata* | *Pustulosa kieneriana* | 6.36 | 2.07 | 46 | **0.018** |  |
|  |  | *Lampsilis ornata* | *Quadrula verrucosa* | 0.78 | 2.07 | 46 | 0.982 |  |
|  |  | *Pustulosa kieneriana* | *Quadrula verrucosa* | -5.58 | 2.07 | 46 | **0.047** |  |
|  | Site 3 | *Fusconaia cerina* | *Lampsilis ornata* | 5.06 | 2.24 | 46 | 0.123 |  |
|  |  | *Fusconaia cerina* | *Pleurobema decisum* | 3.81 | 1.63 | 30 | 0.1627 |  |
|  |  | *Fusconaia cerina* | *Pustulosa kieneriana* | -3.50 | 2.24 | 46 | 0.409 |  |
|  |  | *Fusconaia cerina* | *Quadrula verrucosa* | 0.72 | 1.97 | 46 | 0.983 |  |
|  |  | *Lampsilis ornata* | *Pleurobema decisum* | -1.25 | 1.76 | 30 | 0.9528 |  |
|  |  | *Lampsilis ornata* | *Pustulosa kieneriana* | -8.56 | 2.39 | 46 | **0.005** |  |
|  |  | *Lampsilis ornata* | *Quadrula verrucosa* | -4.33 | 2.14 | 46 | 0.194 |  |
|  |  | *Pleurobema decisum* | *Pustulosa kieneriana* | -7.31 | 1.76 | 30 | **0.0022** |  |
|  |  | *Pleurobema decisum* | *Quadrula verrucosa* | -3.08 | 1.55 | 30 | 0.2941 |  |
|  |  | *Pustulosa kieneriana* | *Quadrula verrucosa* | 4.22 | 2.14 | 46 | 0.213 |  |
|  | Site 8 | *Fusconaia cerina* | *Lampsilis ornata* | 0.09 | 1.85 | 46 | 1.000 |  |
|  |  | *Fusconaia cerina* | *Pleurobema decisum* | 0.94 | 1.55 | 30 | 0.973 |  |
|  |  | *Fusconaia cerina* | *Pustulosa kieneriana* | -11.12 | 1.97 | 46 | **<0.0001** |  |
|  |  | *Fusconaia cerina* | *Quadrula verrucosa* | -1.64 | 2.14 | 46 | 0.87 |  |
|  |  | *Lampsilis ornata* | *Pleurobema decisum* | 0.85 | 1.55 | 30 | 0.9812 |  |
|  |  | *Lampsilis ornata* | *Pustulosa kieneriana* | -11.21 | 1.97 | 46 | **<0.0001** |  |
|  |  | *Lampsilis ornata* | *Quadrula verrucosa* | -1.73 | 2.14 | 46 | 0.849 |  |
|  |  | *Pleurobema decisum* | *Pustulosa kieneriana* | -12.06 | 1.63 | 30 | **<0.0001** |  |
|  |  | *Pleurobema decisum* | *Quadrula verrucosa* | -2.58 | 1.76 | 30 | 0.5916 |  |
|  |  | *Pustulosa kieneriana* | *Quadrula verrucosa* | 9.47 | 2.14 | 46 | **0.001** |  |
| Cirri density | Site 1 | *Fusconaia cerina* | *Lampsilis ornata* | 588 | 224 | 46 | 0.05 |  |
|  |  | *Fusconaia cerina* | *Pustulosa kieneriana* | 1001 | 267 | 46 | **0.003** |  |
|  |  | *Fusconaia cerina* | *Quadrula verrucosa* | -483 | 224 | 46 | 0.15 |  |
|  |  | *Lampsilis ornata* | *Pustulosa kieneriana* | 413 | 254 | 46 | 0.373 |  |
|  |  | *Lampsilis ornata* | *Quadrula verrucosa* | -1071 | 207 | 46 | **<0.0001** |  |
|  |  | *Pustulosa kieneriana* | *Quadrula verrucosa* | -1484 | 254 | 46 | **<0.0001** |  |
|  | Site 2 | *Fusconaia cerina* | *Lampsilis ornata* | 608 | 197 | 46 | **0.017** |  |
|  |  | *Fusconaia cerina* | *Pustulosa kieneriana* | 157 | 197 | 46 | 0.854 |  |
|  |  | *Fusconaia cerina* | *Quadrula verrucosa* | 295 | 197 | 46 | 0.446 |  |
|  |  | *Lampsilis ornata* | *Pustulosa kieneriana* | -451 | 207 | 46 | 0.145 |  |
|  |  | *Lampsilis ornata* | *Quadrula verrucosa* | -313 | 207 | 46 | 0.438 |  |
|  |  | *Pustulosa kieneriana* | *Quadrula verrucosa* | 137 | 207 | 46 | 0.910 |  |
|  | Site 3 | *Fusconaia cerina* | *Lampsilis ornata* | 808 | 224 | 46 | **0.004** |  |
|  |  | *Fusconaia cerina* | *Pleurobema decisum* | -378 | 175 | 30 | 0.221 |  |
|  |  | *Fusconaia cerina* | *Pustulosa kieneriana* | 259 | 224 | 46 | 0.656 |  |
|  |  | *Fusconaia cerina* | *Quadrula verrucosa* | 644 | 197 | 46 | **0.010** |  |
|  |  | *Lampsilis ornata* | *Pleurobema decisum* | -378 | 189 | 30 | 0.221 |  |
|  |  | *Lampsilis ornata* | *Pustulosa kieneriana* | -549 | 197 | 46 | 0.114 |  |
|  |  | *Lampsilis ornata* | *Quadrula verrucosa* | -164 | 214 | 46 | 0.869 |  |
|  |  | *Pleurobema decisum* | *Pustulosa kieneriana* | 637 | 189 | 30 | **0.016** |  |
|  |  | *Pleurobema decisum* | *Quadrula verrucosa* | 1022 | 166 | 30 | **<0.0001** |  |
|  |  | *Pustulosa kieneriana* | *Quadrula verrucosa* | 385 | 224 | 46 | 0.287 |  |
|  | Site 8 | *Fusconaia cerina* | *Lampsilis ornata* | 493 | 185 | 46 | 0.051 |  |
|  |  | *Fusconaia cerina* | *Pleurobema decisum* | -947 | 166 | 30 | **<0.0001** |  |
|  |  | *Fusconaia cerina* | *Pustulosa kieneriana* | -263 | 197 | 46 | 0.544 |  |
|  |  | *Fusconaia cerina* | *Quadrula verrucosa* | -116 | 214 | 46 | 0.949 |  |
|  |  | *Lampsilis ornata* | *Pleurobema decisum* | -1440 | 166 | 30 | **<0.0001** |  |
|  |  | *Lampsilis ornata* | *Pustulosa kieneriana* | -756 | 197 | 46 | **0.002** |  |
|  |  | *Lampsilis ornata* | *Quadrula verrucosa* | -609 | 214 | 46 | **0.032** |  |
|  |  | *Pleurobema decisum* | *Pustulosa kieneriana* | 684 | 175 | 30 | **0.004** |  |
|  |  | *Pleurobema decisum* | *Quadrula verrucosa* | 832 | 189 | 30 | **0.001** |  |
|  |  | *Pustulosa kieneriana* | *Quadrula verrucosa* | 147 | 224 | 46 | 1.000 |  |

# **Figure S1:** a) Scanning electron micrograph of gill latero-frontal cirri (lfc) of *Qudrula verrucosa* from Site 3. b) Scanning electron micrograph of *Lampsilis ornata* gill lfc from Site 2.


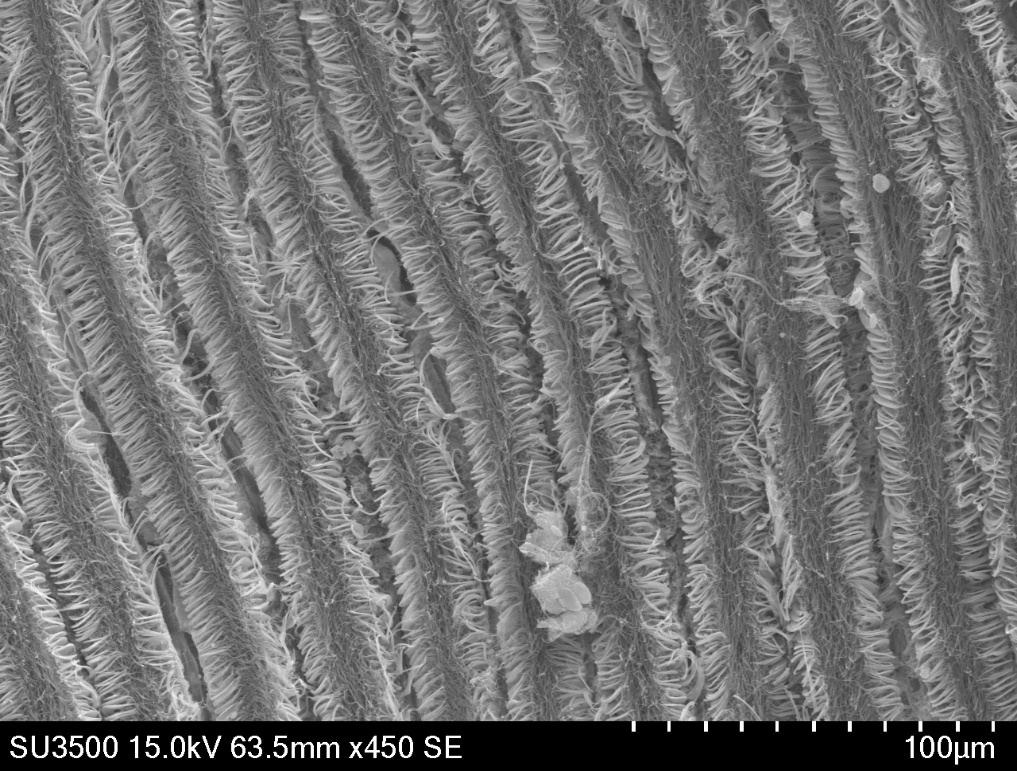

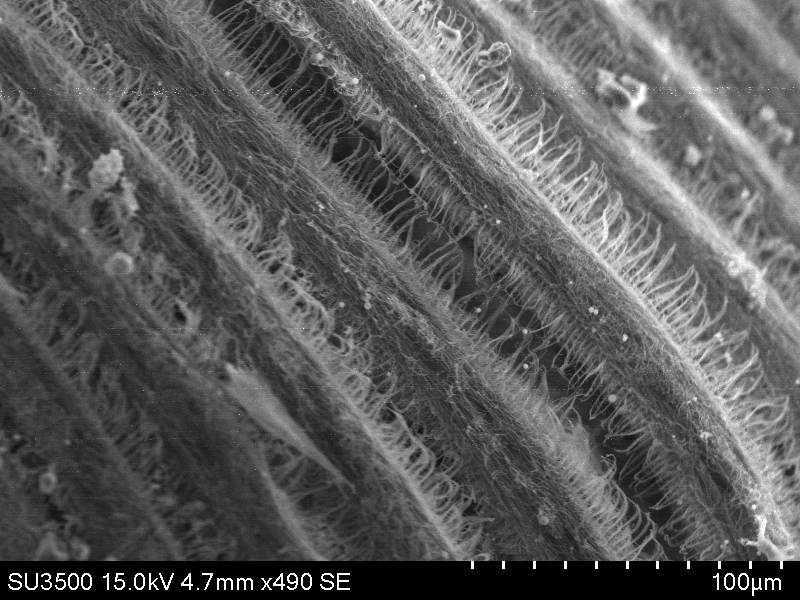


a

b

lfc

lfc

#
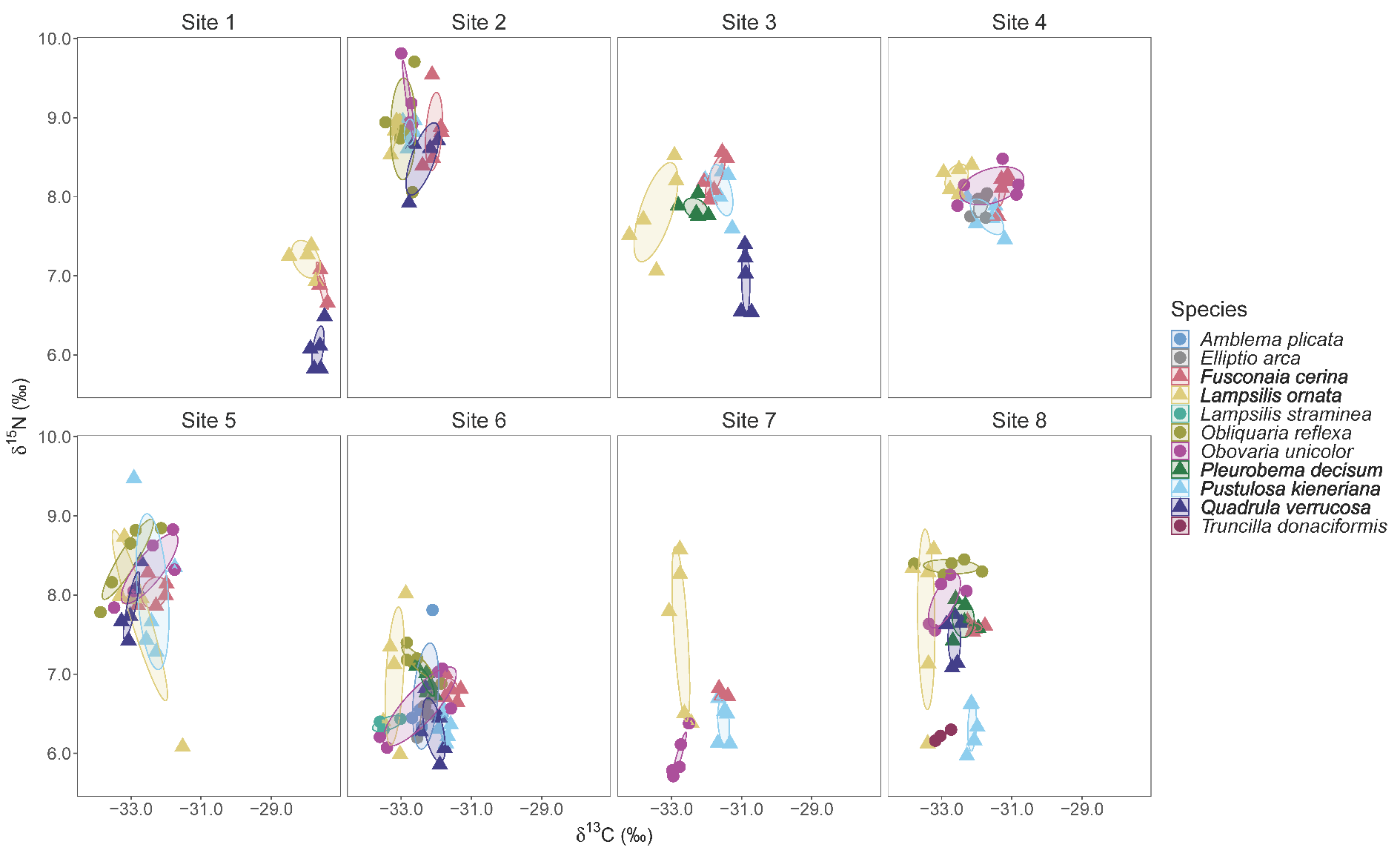
**Figure S2**: Isotope biplots and standard ellipse areas (ellipses are 95% confidence intervals) of 11 mussel species from Site 1 (upstream) to Site 8 (downstream). Bolded names on legend with triangular shaped points are associated with the species that have corresponding gill morphology data. Non-bolded names on legend with circular shaped points do not have morphological data. Scales of *x*- and *y*-axes axes are the same.
